# Stigma, overprotection, and suicidal ideation in epilepsy: a cross-sectional study on psychosocial predictors

**DOI:** 10.1016/j.ebr.2025.100815

**Published:** 2025-08-07

**Authors:** Harun Yetkin, Ozdem Erturk Cetin, Esra Nur Sancar, Inci Su Tascan

**Affiliations:** a Erenkoy Mental and Nervous Diseases Training and Research Hospital, Department of Psychiatry, Istanbul, Turkey; b Sancaktepe Sehit Prof. Dr. Ilhan Varank Training and Research Hospital, Department of Neurology, Istanbul, Turkey

**Keywords:** Epilepsy, Stigma, Overprotection, Suicidal ideation, Depression, Suicide probability

## Abstract

Patients with epilepsy (PWE) are at elevated risk for suicide, with psychosocial factors such as felt stigma and perceived overprotection contributing to this vulnerability. This cross-sectional study examined the associations among suicidal ideation, felt stigma, and perceived overprotective behaviors in 92 adult PWE not currently receiving psychiatric treatment. Participants completed self-report measures including the Felt Stigma Scale (FSS), Perceived Overprotection Scale (POS), Beck Depression Inventory-II (BDI-II), and Suicide Probability Scale (SPS). SPS scores were significantly correlated with FSS (r = 0.405, p < 0,001) and BDI-II (r = 0.765, p < 0.001), while FSS and POS were also correlated (r = 0.399, p < 0.001). POS was not directly associated with SPS. In hierarchical regression, FSS was a significant predictor of suicidal ideation (p < 0.001), but BDI-II emerged as the strongest predictor (p < 0.001), explaining 58 % of the variance in SPS scores. These findings highlight the mediating role of depression in the relationship between felt stigma and suicidal ideation, and suggest that addressing felt stigma and depressive symptoms in epilepsy care may help reduce suicide risk.

## Highlights

• Felt stigma is significantly associated with suicidal ideation in epilepsy.
• Depression mediates the effect of stigma on suicide risk among people with epilepsy.
• Perceived overprotection correlate with stigma but not directly with suicidality.
• This is the first study linking stigma, overprotection, and suicidality in epilepsy.
• Addressing psychosocial factors may help reduce suicide risk in epilepsy care.

## 1 Introduction

Epilepsy is a chronic neurological disease that affects more than 70 million people worldwide and imposes a substantial psychological and social burden [1]. Patients with epilepsy (PWE) often experience difficulties in social integration, education, and employment, which can lead to reduced quality of life, low self-esteem, and mental health disorders [2,3]. In addition to the physical burden of seizures, the unpredictable nature of epilepsy often leads to heightened vigilance and perceived threat, contributing to significant psychosocial distress [4]. Studies suggest that PWE have a shorter lifespan than the rest of the population and a 2-fold higher risk of completed suicide [5,6]. Several psychiatric disorders, particularly depression, anxiety, and suicidal ideation, have been frequently reported in this population [[7], [8], [9], [10]].

Stigma plays a crucial role in shaping the psychosocial experience of PWE. Stigma in epilepsy includes internalized, perceived, and enacted components, all of which may negatively affect emotional well-being [11]. Felt stigma, defined as the internal perception of being socially devalued, is associated with increased depressive symptoms and suicidal ideation [12,13]. As assessed in this study, felt stigma corresponds to internalized stigma within this widely accepted framework. Previous studies have shown that felt stigma among PWE is significantly correlated with depression, anxiety, low self-esteem, and poor quality of life [[14], [15], [16], [17]]. Furthermore, felt stigma may contribute to social withdrawal, secrecy, and reduced treatment compliance, exacerbating psychological vulnerability [18,19].

Overprotective behaviors from family members may also contribute to emotional distress in PWE. Overprotection is often well-intentioned but may restrict autonomy and reinforce dependency, particularly in collectivist cultures where familial involvement is high [20,21]. Excessive parental control and overprotection have been associated with higher levels of depression and lower levels of resilience in individuals with chronic conditions [22,23]. In epilepsy, perceived overprotection may foster feelings of inadequacy, reinforce internalized stigma, and increase vulnerability to mood disorders [24,25].

While several studies have examined the impact of felt stigma and depressive symptoms on suicidal ideation, the role of perceived overprotection remains underexplored. This study aims to investigate the psychosocial predictors of suicidal ideation in PWE, with particular focus on felt stigma, perceived overprotection, and depressive symptoms. We hypothesized that both felt stigma and perceived overprotection would be positively associated with suicidal ideation, and that depressive symptoms would mediate these relationships.

## 2 Methods

### 2.1 Participants

This study was conducted with patients diagnosed with epilepsy who were being followed in the Epilepsy Outpatient Clinic of the Neurology Department at a tertiary university hospital in Turkey. A total of ninety-two patients participated in the study. All participants had no other neurological diagnoses besides epilepsy. Inclusion criteria required participants to be over 18 years of age, literate, and without any known major psychiatric disorders (such as bipolar affective disorder, schizophrenia/psychosis, or intellectual disability), and not receiving any psychiatric treatment. No specific duration of epilepsy diagnosis was stipulated. All participants were evaluated by a neurologist through face-to-face consultations. Patients with brain tumors, infections, metabolic disorders, or a diagnosis of psychogenic non-epileptic seizures were excluded from the study. Written informed consent was obtained from all participants in accordance with the Declaration of Helsinki. Patients were informed about the study’s scope and were asked to complete the Sociodemographic Data Form, the Felt Stigma Scale, the Perceived Overprotection Scale, the Beck Depression Inventory-II, and the Suicide Probability Scale. Patients identified as high suicide risk or with severe depressive symptoms during the assessment were referred to psychiatric outpatient clinics for further evaluation and treatment in accordance with institutional protocols. This study was approved by the Ethics Committee of Sancaktepe Ilhan Varank Hospital on November 10, 2023, under protocol number 2023/228.

### 2.2 Questionnaires

#### 2.2.1 Sociodemographic Scale

The questionnaire consisted of twelve questions in total. Seven were multiple-choice items assessing gender, marital status, education level, occupation, and living situation. Five were open-ended questions regarding medical conditions and psychiatric history. Patients were asked whether they had ever received psychiatric treatment, and if so, what treatment they received and for what reason. In order to en sure diagnostic clarity, participants were only included if there was no clinical indication of major neurological (e.g., intracranial mass, encephalitis) or psychiatric disorders (e.g., psychosis, bipolar disorder, intellectual disability). These criteria were applied to reduce the risk of confounding effects. Patients with stable chronic conditions such as diabetes or hypertension were included, as these were not expected to influence the psychiatric outcomes.

#### 2.2.2 Felt stigma scale

This scale was developed by Baybaş et al. to assess felt stigmatization in PWE for Turkish population, focusing on five main domains: discrimination, stigma resistance, false beliefs, social isolation, and inadequacy, and it is a self-report instrument (Supplementary 1). The questionnaire contains 32 items rated on a 4-point Likert scale: 1 (absolutely disagree), 2 (disagree), 3 (agree), and 4 (absolutely agree). Scores range from 25 to 100, with a cut-off point of 50. Patients were categorized into three groups: 25–50 = not stigmatized, 51–75 = moderately stigmatized, and 76–100 = highly stigmatized [12]. The Cronbach’s alpha value for the scale was 0.915.

#### 2.2.3 Beck depression Inventory-II

This scale is widely used to assess depressive symptoms and has frequently been applied in epilepsy research. It consists of 21 items rated on a 4-point Likert scale ranging from 0 to 3, and patients are asked to evaluate their emotional state over the previous 14 days [13]. Scores between 11 and 15 indicate mild depression, 16–23 moderate depression, and scores above 24 suggest severe depression [14]. In this study, the BDI-II was used as a correlational measure in relation to suicidal ideation. A cutoff score of ≥13 on the BDI-II was used to define clinically significant depressive symptoms, based on the Turkish validation study by Kapcı et al. [15].

#### 2.2.4 Suicide probability scale

This scale was originally developed by Cull and Gill and is a self-report measure assessing current suicide risk and behavior [16]. It includes 36 items rated from 1 to 4, with response options ranging from “none of the time” to “all of the time.” It comprises 12 items measuring hopelessness, 8 for suicidal ideation, 9 for self-negative evaluation, and 7 for hostility. The Cronbach’s alpha coefficient for the scale was 0.93. In the present study, SPS scores were correlated with the BDI-II.

#### 2.2.5 The perceived overprotection scale

This scale, developed by Aydemir, evaluates the perception of parental overprotection in Turkish PWE. It contains ten items rated on a 5-point Likert scale, where 5 indicates “completely agree” and 1 indicates “completely disagree.” Items such as “My family never lets me go out alone” and “I think my family gives me less responsibility than I am capable of managing because of my epilepsy” assess autonomy, independence, and perceived competence. The internal consistency coefficient of the scale was 0.85 [10].

### 2.3 Research design

This cross-sectional, single-center study was conducted between November 2023 and April 2024. A total of 102 patients were initially recruited from the Neurology Outpatient Clinic at Sancaktepe Ilhan Varank Hospital. All patients who agreed to participate in the study were included. However, two patients with intellectual disabilities, one patient with a diagnosis of psychosis, four illiterate patients, and three patients with incomplete form submissions were excluded, yielding a final sample of 92 participants. All participants signed informed consent before inclusion. A neurologist conducted interviews during routine visits and distributed the questionnaires. Participants completed the forms independently and returned them to their physicians. The completed forms were later evaluated by psychiatrists in this study.

### 2.4 Statistical analysis

Statistical analyses were conducted using the Statistical Package for the Social Sciences (SPSS), version 26.0. Descriptive statistics, including frequencies, percentages, means, and standard deviations, were used to summarize demographic and clinical characteristics. Pearson correlation analyses were performed to examine associations between felt stigma, perceived overprotection, depression, and suicidal ideation. Hierarchical linear regression analyses were used to explore the predictive value of these variables for suicide probability scores. A p-value < 0.05 was considered statistically significant. Normality of data was assessed using skewness and kurtosis values (acceptable range: ±1.5). Additionally, a mediation analysis was conducted using Hayes’ PROCESS macro (version 4.2, Model 4) for SPSS (Hayes, 2022). Depression was defined as the independent variable, suicide probability as the dependent variable, and felt stigma and perceived overprotection as parallel mediators. The indirect effects were tested using the bootstrap method with 5000 resamples. Statistical significance was determined based on whether the 95 % bias-corrected confidence intervals excluded zero.

## 3 Results

The average age of the participants was 32.18 ± 10.64 years. Among those evaluated in the study, 60 (65,2%) were female. Regarding marital status, 43 (46.7 %) were single, 48 (52.2 %) were married, and 1 (1.1 %) was divorced. In terms of educational background, 2 (2.2 %) were literate but had never attended school, 22 (23.9 %) had completed primary education, 37 (40.2 %) had graduated from high school, and 31 (33.7 %) had attained higher education or beyond. Living arrangement data showed that 3 (3.3 %) lived alone, 42 (45.7 %) lived with their parents, 44 (47.8 %) lived with a spouse and children, and 3 (3.3 %) had other living arrangements. Regarding employment status, 43 (46.7 %) were employed, 47 (51.1 %) were unemployed, and 2 (2.2 %) were retired (Table 1).

**Table 1:** Demographic characteristics of the cases evaluated in the study (n: number, %: percentage).

		N = 92	%
Gender	Female	60	65,2
	Male	32	34.8
Marital Status	Single	43	46.7
	Married	48	52.2
	Divorced	1	1.1
Education	Homeschooled	2	2.2
	Primary School graduate	22	23.9
	High School graduate	37	40.2
	University	31	33.7
Living Situation	Alone	3	3.3
	With parents/guardians	42	45.7
	Significant other/children	44	47.8
	Other	3	3.3
Working Status	Working	43	46.7
	Not Working	47	51.1
	Retired	2	2.2
Other medical conditions	None	49	53.3
	Yes	43	46.7

Among the 92 patients included in the study, 2 individuals (2.17 %) scored above 110 on the Suicide Probability Scale (SPS), indicating a high risk of suicidal ideation. Regarding felt stigma, 43 patients (46.7 %) reported moderate levels, while 9 patients (9.8 %) reported highly stigmatized. In total, 56.5 % of the participants experienced at least moderate felt stigma. The mean score on the Perceived Overprotection Scale (POS) was 26.91. Based on the BDI-II, 18 patients (19.6 %) showed signs of moderate depression, and 10 patients (10.9 %) had severe depressive symptoms. The mean BDI-II score across the sample was 15.91.

According to Pearson correlation analysis, a statistically significant positive correlation was found between the scores of the Suicide Probability Scale (SPS) and the Felt Stigma Scale (FSS) (r = 0.405, p < 0.001). Suicidal ideation scores and depression scores, as demonstrated by previous studies, are strongly linked (r = 0.765, p < 0.001). Furthermore, Pearson correlation analysis revealed a statistically significant positive correlation between felt stigma and depression scores (r = 0.439, p < 0.001), as well as between felt stigma and perceived overprotection scores (r = 0.399, p < 0.001) (Table 2).

**Table 2:** Relationship between Suicide Probability, Felt Stigma, Beck Depression and Perceived Overprotection scores (r: Pearson Correlation Analysis, p: Statistical significance).

		SPS	FSS	BDI-II
Suicide Probability Scale (SPS)	r	−	−	
	p	−	−	
Felt Stigma Scale (FSS)	r	0.405	−	
	p	<0.001	−	
Beck Depression Inventory-II (BDI-II)	r	0.765	0.439	
	p	<0.001	<0.001	
Percieved Overprotection Scale (POS)	r	0.143	0.399	0.160
	p	0.174	<0.001	0.129

According to the Hierarchical Regression Analysis, 16 % of the variance in SPS scores was statistically significantly explained by FSS scores (F = 17.65, p < 0.001). When POS scores were added to this model, 15 % of the variance in SPS scores was statistically significantly explained by both FSS and POS scores (F = 8.75, p < 0.001). However, our analysis revealed that only FSS scores (p < 0.001, β: 0.164–0.502) had a statistically significant effect on explaining SPS scores independently (see Table 3).

**Table 3:** Effectiveness of FSS, BDI-II, and POS Scores in Explaining SPS Scores (SE=Standard Error, R^2^ = coefficient of determination).

	Modal	Unstandardized Coefficients		Standardized Coefficients				
		B	SE	β	t	p	R^2^	F
	Constant	54.939	4.519		12.156	<0.001		
	Felt Stigma Scale (FSS)	0.326	0.078	0.405	4.201	<0.001	16	17.65
	Constant	55.339	4.927		11.232	<0.001		
	Felt Stigma Scale (FSS)	0.333	0.085	0.414	3.915	<0.001	15	8.75
	Percieved Overprotection Scale (POS)	−0.030	0.141	−0.022	−0.210	0.834		
	Constant	56.916	3.468		16.413	<0.001		
	Felt Stigma Scale (FSS)	0.071	0.066	0.089	1.085	0.281		
	Percieved Overprotection Scale (POS)	−0.011	0.099	−0.009	−0.116	0.908	0.58	42.49
	Beck Depression Inventory-II (BDI-II)	0.788	0.082	0.728	9.594	0<.001		

With the addition of BDI-II scores, 58 % of the variance in SPS scores was statistically significantly explained by FSS, POS, and BDI-II scores (F = 42.49, p < 0.001). This finding highlights that depression is a key factor in explaining suicidal ideation (p < 0.001, β: 0.625–0.951). For the sake of clarity in this research, we also examined the correlation between felt stigma (FSS) scores and both perceived overprotection (POS) and depression (BDI-II) scores. It was found that 16 % of the variance in FSS scores was statistically significantly explained by POS scores (F = 17.65, p < 0.001). When BDI-II scores were added, 30 % of the variance in FSS scores was statistically significantly explained by both POS and BDI-II scores (F = 19.44, p < 0.001). Both POS (p < 0.001, β: 0.265–0.855) and BDI-II (p < 0.001, β: 0.279–0.757) scores had statistically significant effects on explaining FSS scores (Table 4).To further explore potential group differences, we conducted additional analyses comparing participants with and without clinically significant depressive symptoms (BDI-II ≥ 13). Results are presented in Table 5, Table 6.

**Table 4:** Effectiveness of BDI-II and POS Scores in Explaining FSS Scores (SE=Standard Error, R^2^ = coefficient of determination).

Modal		Unstandardized coefficients		Standardized coefficients				
		B	SE	β	t	p	R^2^	F

	Constant	37.957	4.605		8.243	<0.001		
	Percieved Overprotection Scale (POS)	0.662	0.160	0.399	4.129	<0.001	16	17.05
	Constant	32.458	4.403		7.372	<0.001		
	Percieved Overprotection Scale (POS)	0.560	0.149	0.338	3.769	<0.001	0.30	19.44
	Beck Depression Inventory-II (BDI-II)	0.518	0.120	0.385	4.303	<0.001

**Table 5:** Comparison of demographic and clinical characteristics based on depression status. M = mean, SD=Standard Deviation, X^2^=Chi-Square Test, t = Independent Sample t-Test.

		BDI-II < 13		BDI-II ≥ 13			
		n/M	%/SD	n/M	%/SD	Analysis	p
Age		33	10	31	11	t = 0.661	0.510
Gender	Female	25	56.8	35	72.9	X^2^ = 2.623	0.105
	Male	19	43.2	13	27.1		
Marital Status	Single	17	38.6	26	54.2	X^2^ = 3.049	0.218
	Married	26	59.1	22	45.8		
	Divorced	1	2.3	0	0.0		
Eduaction	Homeschooled	0	0.0	2	4.2	X^2^ = 2.288	0.515
	Primary School	12	27.3	10	20.8		
	High School	17	38.6	20	41.7		
	University	15	34.1	16	33.3		
Living Situation	Alone	2	4.5	1	2.1	X^2^ = 2.111	0.550
	With parents/guardians	17	38.6	25	52.1		
	Significant other	23	52.3	21	43.8		
	Other	2	4.5	1	2.1		
Working Status	Working	23	52.3	20	41.7	X^2^ = 1.080	0.583
	Not Working	20	45.5	27	56.3		
	Retired	1	2.3	1	2.1		
Other Medical Conditions	None	26	59.1	25	52.1	X^2^ = 0.456	0.499
	Yes	18	40.9	23	47.9		
Previous Psychiatric Treatment	Yes	8	18.2	21	43.8	X^2^ = 6.953	0.008
	No	36	81.8	27	56.3

**Table 6:** Comparison of suicide, epilepsy stigma, and total overprotection scores according to depression status. M = mean, SD=Standard Deviation, t = Independent Sample t-Test.

	BD-II < 13		BDI-II ≥ 13			
	n/M	%/SD	n/M	%/SD	t	p
Total Suicide Probability Scale (SPS)	66.66	8.53	79.06	14.52	−4.937	<0.001
Total Felt Stigma Scale (FSS)	49.43	13.66	61.58	17.34	−3.711	<0.001
Total Percieved Overprotection Scale (POS)	24.45	9.01	29.17	10.60	−2.286	0.025

In the Chi-square analysis, participants with clinically significant depressive symptoms were significantly more likely to have a history of psychiatric treatment (χ^2^ = 6.953, p = 0.008). No other sociodemographic or clinical variables differed significantly between the two groups (p > 0.05).

In the independent samples *t*-test, participants with depressive symptoms had significantly higher mean scores on the Suicide Probability Scale (t = -4.937, p < 0.001), the Felt Stigma Scale (t = -3.711, p < 0.001), and the Perceived Overprotection Scale (t = -2.286, p = 0.025), compared to those without depressive symptoms.

**Fig. 1:**
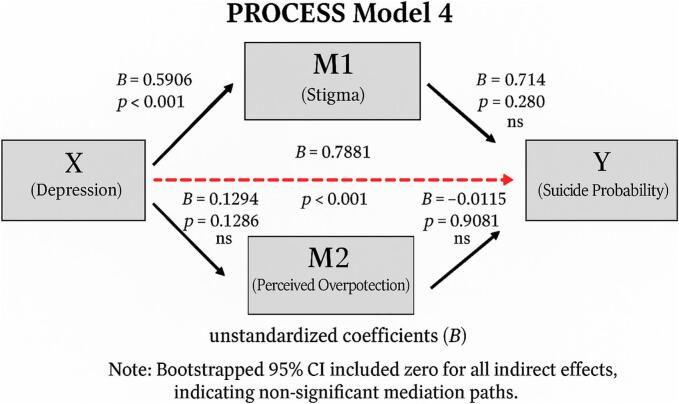
PROCESS Model 4 results illustrating the parallel mediating roles of felt stigma (M_1_) and perceived overprotection (M_2_) in the relationship between depression (X) and suicidal ideation (Y). Unstandardized coefficients (B) and corresponding p-values are presented. All indirect effects were statistically non-significant.

Finally, a mediation analysis was conducted to explore whether felt stigma and perceived overprotection mediated the relationship between depressive symptoms and suicidal ideation. The results are presented in Fig. 1.

As shown in the model, depressive symptoms significantly predicted suicidal ideation (B = 0.79, p < 0.001). However, neither felt stigma (indirect effect = 0.042, 95 % CI [–0.028, 0.161]) nor perceived overprotection (indirect effect = –0.002, 95 % CI [–0.031, 0.036]) significantly mediated this relationship. The total indirect effect was not statistically significant (0.041, 95 % CI [–0.028, 0.163]).

## 4 Discussion

This study investigated the relationships among felt stigma, perceived overprotection, and suicidal ideation in PWE. We hypothesized that both felt stigma and perceived overprotection would be associated with increased suicidal ideation. While our results confirmed the well-established association between depression and suicidality—consistent with previous research [17]—they only partially supported our hypothesis. Specifically, although felt stigma was significantly associated with depressive symptoms, it did not independently predict suicidal ideation.

Suicidality is a recognized comorbidity in epilepsy, with population-based studies reporting a lifetime prevalence of suicidal ideation of up to 25 % [4]. Tellez-Zenteno et al. [18] found that adults with epilepsy were 2.2 times more likely to report suicidal ideation than individuals without epilepsy. Similarly, a study conducted in West China with over 2,000 epilepsy patients identified both depression and stigma as critical psychological correlates of suicide risk [19]. Our findings are consistent with this literature, highlighting depression as the strongest predictor of suicide risk in this population. The robust association between depression and suicidality is well-established in the psychiatric literature, with major depressive disorder implicated in up to 60 % of suicide deaths [20].

Stigma remains a significant psychosocial burden for many PWE [20]. Multiple studies have reported high levels of felt stigma in this population [22,23]. A large-scale European study involving more than 6,000 participants found that nearly half struggled to accept their condition, and 17 % reported direct experiences of stigma. A web-based Norwegian survey found that 56 % of respondents reported feeling stigmatized, and 35 % had experienced discrimination due to epilepsy. Overall, 70 % of participants endorsed at least one type of stigma. These findings align with our results and further support the consistent association between felt stigma and depressive symptoms [[24], [25], [26]]. Moreover, recent evidence suggests that depression itself may be a predictor of increased stigma internalization [26]. These bidirectional associations suggest that targeting felt stigma may help alleviate depressive symptoms and reduce overall psychological distress. However, in our model, felt stigma did not significantly mediate the relationship between depression and suicidal ideation. These results highlight the complex interplay among stigma, family dynamics, and emotional distress, even in the absence of statistically significant mediation pathways. Our findings suggest that higher levels of felt stigma may be associated with increased perceptions of overprotection. While both constructs were correlated with suicidal ideation, neither significantly mediated the relationship between depression and suicidality in our mediation model.

Our study also found significant correlations among felt stigma, depressive symptoms, and overprotective family behavior. Previous literature has demonstrated that overprotection is associated with concerns about future autonomy, social functioning, and self-image in PWE [10]. Overprotective family dynamics may unintentionally promote dependency and low self-efficacy, thereby contributing to internalized stigma. PWE often report perceiving themselves as less competent or less valuable than their peers, and overprotective caregiving may reinforce these perceptions [27].

For example, while the majority of our participants were married—contrary to previous studies that often report higher rates of unmarried status among PWE—high levels of stigma and emotional burden were still observed [2,28]. This discrepancy may reflect traditional cultural norms around marriage in Turkey, where marital status does not necessarily buffer against psychosocial distress. Similarly, although the educational profile of our sample was generally consistent with national averages, lower educational attainment has been repeatedly associated with increased stigma, overprotection, and emotional vulnerability in the literature [2,28,29]. These patterns may help explain the observed associations among overprotection, depressive symptoms, and stigma-related burden in our study. Coping strategies such as secrecy, fear-based restrictions, and social withdrawal may further exacerbate emotional distress and reinforce stigmatization [30].

Overprotective parenting is often a response to heightened parental stress and anxiety regarding the child’s health. Research shows that parents of children with epilepsy frequently provide excessive support to the affected child, while inadvertently neglecting the needs of their other children [31]. Families that combine high levels of control with an indulgent style often restrict autonomy development and promote psychological dependency [32]. Although such strategies may be well-intentioned, they often lead to increased isolation and reinforce stigma-related cognitions.

To further explore the pathways linking psychosocial stressors and suicidal ideation, a mediation analysis was conducted using depression as the independent variable, suicide probability as the outcome, and felt stigma and perceived overprotection as parallel mediators. The analysis revealed that depression had a strong direct effect on suicide risk. However, neither felt stigma nor perceived overprotection significantly mediated this relationship. These results suggest that while stigma and overprotection are psychosocial burdens for PWE, their impact on suicidality may not operate through indirect pathways involving depression. Rather, these factors may independently contribute to emotional distress, emphasizing the multifactorial nature of suicide risk in this population.

While overprotection is often motivated by a desire to protect individuals from social harm, it may paradoxically contribute to poorer psychosocial functioning. A study has shown that reduced perceived overprotection is associated with increased employability among PWE [33]. Excessive protection can erode self-confidence and hinder social integration, indirectly reinforcing feelings of stigmatization. Our findings suggest that perceived overprotection, while not a direct predictor of suicidal ideation, remains clinically relevant due to its association with increased emotional distress and stigma. It should be noted that both the BDI-II and SPS include items assessing suicidal ideation. Although the SPS was chosen to provide a broader assessment of suicide risk, this item-level overlap may have contributed to inflated correlations between depression and suicidality. This potential redundancy should be considered when interpreting the observed associations.

In supplementary analyses, participants with clinically significant depressive symptoms (BDI-II ≥ 13) exhibited significantly higher levels of suicidal ideation, felt stigma, and perceived overprotection. These findings further support the central role of depression as a primary driver of suicidality in PWE, particularly among those experiencing higher levels of stigma and overprotection. Moreover, participants with depressive symptoms were more likely to have a history of psychiatric treatment, although other demographic or clinical characteristics did not differ significantly. These patterns highlight the psychosocial vulnerability of this subgroup and underscore the need for integrated mental health support in epilepsy care.

Although validated instruments were used to assess psychological constructs, all data were obtained via self-report questionnaires. This methodology may have introduced certain response biases, particularly underreporting and social desirability effects. These limitations are especially relevant when assessing sensitive experiences such as suicidal ideation. Therefore, findings should be interpreted with caution, and future studies may benefit from incorporating structured clinical assessments to enhance diagnostic accuracy and validity.

## 5 Limitations

This study has several limitations that should be acknowledged. First, the absence of a control group limits the ability to draw causal inferences and reduces the generalizability of the findings to non-epileptic populations. Second, participants were recruited from a single tertiary care center, which may restrict the representativeness of the sample. Third, the study relied exclusively on self-report measures and did not include structured psychiatric interviews, increasing the likelihood of undetected subthreshold psychopathology. Furthermore, clinical variables such as epilepsy type, seizure frequency, illness duration, and antiepileptic drug regimen were not systematically assessed. Although these variables are clinically important, they were excluded due to difficulties in obtaining consistent and complete clinical data across participants. Many patients had fragmented follow-up histories across different healthcare settings, and medical documentation was often unavailable or incomplete. As a result, reliable retrospective collection or verification of epilepsy-related clinical information was not feasible. Future research should aim to address these limitations by incorporating multi-center samples, objective clinical data, and longitudinal designs to better elucidate the complex psychosocial mechanisms underlying suicide risk in epilepsy. The lack of detailed epilepsy-related clinical information, including seizure type, frequency, and antiepileptic drug regimens, represents a significant limitation and may affect the interpretation of psychosocial correlates observed. This limitation should be carefully considered when interpreting the findings and highlights the need for future studies with comprehensive clinical data collection. Additionally, no follow-up data were collected for participants who were referred for psychiatric evaluation due to high suicide risk or severe depressive symptoms. This lack of outcome monitoring represents an ethical limitation that should be acknowledged.

## 6 Conclusion

This study underscores the indirect but critical role of felt stigma in increasing suicidal ideation among PWE, primarily through its strong association with depressive symptoms. Although perceived overprotection was not directly linked to suicide risk, it may reinforce internalized stigma and emotional distress, thereby contributing to vulnerability. Routine assessment of stigma and depression, along with psychoeducational interventions targeting both patients and families, may play a preventive role in mitigating suicide risk. Integrating these psychosocial dimensions into the standard care of epilepsy should be considered a key component of holistic and effective disease management.

## Ethical statement

Ethical approval for this study was obtained from Sancaktepe Sehit Prof. Dr. Ilhank Varank Hospital on November 10, 2023, with a 2023/228 identification number. Written informed consent was obtained for anonymized patient information to be published in this article. The Authors declare that there is no conflict of interest. This research received no specific grant from any funding agency in the public, commercial, or not-for-profit sectors.

## CRediT authorship contribution statement

**Harun Yetkin:** Writing – original draft, Project administration, Methodology, Formal analysis, Data curation, Conceptualization. **Ozdem Erturk Cetin:** Writing – review & editing. **Esra Nur Sancar:** Data curation. **Inci Su Tascan:** Writing – original draft, Methodology, Investigation, Data curation.

## Declaration of competing interest

The authors declare that they have no known competing financial interests or personal relationships that could have appeared to influence the work reported in this paper.
