# Supplementary material for: Stigma, overprotection, and suicidal ideation in epilepsy: a cross-sectional study on psychosocial predictors
Source: Epilepsy Behav Rep. 2025 Aug 7;33:100815. doi: 10.1016/j.ebr.2025.100815 (PMC13031039; doi:10.1016/j.ebr.2025.100815)
Supplement: Supplementary Data 1 [file mmc1.docx]

1. Associated Data

This section contains any data availability statements or supplementary materials included in this article.

- 1. Supplementary Materials

1. Felt Stigma Scale

| Patients |
| --- |
| Social isolation |
| 7 I think I have epilepsy as a punishment from God. |
| 9 As I have epilepsy, people don't take me seriously or ignore me. |
| 10 As I have epilepsy, people don't want to be in close relationship with me. |
| 11 As I have epilepsy, I don't think that I would be successful in school and/or at work. |
| 15 As I have epilepsy, I am ashamed. |
| 16 Having epilepsy results in disappointment. |
| 19 As I have epilepsy, I don't think that I could make a contribution to society. |
| 23 I feel alienated due to the negative feelings about epilepsy in society |
| 24 I keep away from events, as I don't want my family and friends to be ashamed. |
| 25 I am not accepted by others of the same age. |
| 27 I don't think that I could be an adequate and good parent for my child. |
| 29 I would prefer to have a disease other than epilepsy. |
| Discrimination |
| 5 Onions, water and cologne are beneficial for stopping epileptic convulsions. |
| 6 Epilepsy is an absolutely untreatable disease. |
| 8 I think that other people discriminate because I have epilepsy. |
| 12 I am different from other people as I have epilepsy. |
| 13  Having epilepsy make me feel inadequate. |
| 14  People who don't have epilepsy can't understand me |
| 22 Men with epilepsy can't become soldiers. |
| 28 When applying for a job, the chance of being accepted is higher for a person  who doesn't have epilepsy. |
| 30 I avoid being in relationships with other people because I think that I will be  rejected as I have epilepsy. |
| Insufficiency |
| 17  When people look at me, they can immediately understand that I have epilepsy. |
| 18  As I have epilepsy, I need others to make decisions for me. |
| 20  People with epilepsy can't get married. |
| 21  Women with epilepsy can't give birth. |
| 26 As I have epilepsy, I can't get along with my relatives. |
| False beliefs |
| 1  Epilepsy is a contagious disease. |
| 2  Epilepsy is a mental disease. |
| 3  Epilepsy is possession. |
| 4  Epilepsy occurs due to fear. |
| Stigma resistance |
| 31  I can live a good life, despite having epilepsy. |
| 32  I feel good when I am together with other people who have epilepsy. |

Supplementary 1. The Felt Stigma Scale in patients and factor analyses, developed by S. Baybas et al.
